# Endophytic Bioactive Compounds for Wound Healing: A Review of Biological Activities and Therapeutic Potential

**DOI:** 10.3390/microorganisms13071691

**Published:** 2025-07-18

**Authors:** Octavio Calvo-Gomez, Farkhod Eshboev, Kamilla Mullaiarova, Dilfuza Egamberdieva

**Affiliations:** 1 Medical School, Central Asian University, Tashkent 111221, Uzbekistan; quarkazul@gmail.com; 2 Faculty of Land Resources, National Research University TIIAME, Tashkent 100000, Uzbekistan; 3 Institute for Advanced Studies, New Uzbekistan University, Tashkent 100007, Uzbekistan; farkhod.eshboev@gmail.com; 4 S. Y. Yunusov Institute of the Chemistry of Plant Substances, Academy of Sciences of Uzbekistan, Tashkent 100170, Uzbekistan; 5 Faculty of Medicine, Tashkent State Medical University, Tashkent 100109, Uzbekistan; camilla.mullaiarova@gmail.com

**Keywords:** endophytes, secondary metabolites, GRADE assessment, tissue regeneration

## Abstract

Endophytic microorganisms inhabiting plant tissues constitute a unique and largely untapped reservoir of bioactive metabolites, including phenolics, terpenoids, alkaloids, polysaccharides, and anthraquinones, among others. This review focuses on the potential of these compounds to modulate the complex processes of wound repair, such as hemostasis, inflammation, proliferation, and remodeling. Uniquely, this review delineates the specific mechanisms supported not only by indirect evidence but by primary research directly linking endophytic metabolites to wound repair. We synthesized and evaluated evidence from 18 studies, of which over 75% directly assessed wound healing effects through in vitro and in vivo models. Metabolites from endophytic microorganisms promoted wound contraction, suppressed biofilm formation by key pathogens (e.g., MRSA, P. aeruginosa), and accelerated tissue re-epithelialization in animal models. Other compounds demonstrated >99% wound closure in rats, while several extracts showed anti-inflammatory and cytocompatible profiles. Nevertheless, the majority of studies applied unstandardized methods and used crude extracts, hindering precise structure–activity assessment. The originality of this review lies in drawing attention to direct evidence for wound healing from diverse endophytic sources and systematically identifying gaps between preclinical promise and clinical translation, positioning endophytes as a sustainable platform for next-generation wound therapeutics.

## 1. Introduction

Wound healing in humans is a complex biological process that involves a coordinated response to tissue injury. It restores tissue integrity and provides a barrier against microbial invasion and environmental factors. As reviewed by Guo and DiPietro (2010) and Wilkinson and Hardman (2020), wound healing involves a dynamic, overlapping cascade of regulated cellular and molecular events: hemostasis, inflammation, proliferation, and remodeling. These phases are mediated by paracrine signals, matrix cues, and direct cell–cell interactions. The process can be disrupted by systemic, local, and environmental factors, including microbial contamination, diabetes, and poor blood perfusion, among others, which may lead to impaired healing, chronic wounds, or excessive scarring [1,2,3].

Wound closure initiates with hemostasis via platelet activation and coagulation cascades. Platelets adhere to exposed extracellular matrix (ECM), release granule contents promoting fibrin clot formation, and secrete chemotactic factors recruiting inflammatory cells. A controlled inflammatory response follows, balancing host defense and potential tissue damage. Neutrophils and subsequently macrophages predominate early, clearing debris, providing antimicrobial defense, and modulating repair through cytokine and growth factor release [3,4]. Following decontamination, the proliferative phase begins, characterized by granulation tissue formation, i.e., fibroblast activation, collagen deposition, and angiogenesis [3,5]. Impaired angiogenesis is associated with wound chronicity. The final remodeling phase involves ECM maturation and cellular apoptosis, resulting in scar tissue with altered mechanical properties but providing barrier function [6,7].

Disruptions in wound healing carry significant clinical consequences. Chronic wounds (e.g., diabetic ulcers, pressure sores, venous leg ulcers) represent a substantial healthcare burden, particularly with aging populations and increased metabolic disease prevalence, associated with high medical costs and reduced quality of life [8,9,10]. Pathogenesis often involves persistent inflammation, biofilm formation, ischemia, or altered cellular phenotypes impairing repair [2,3]. Conversely, excessive repair can lead to hypertrophic scarring or keloid formation [2,3]. Thus, there is a need for therapeutics that modulate healing, resolve infection, and address the wound microenvironment.

The use of natural products for wound management is deeply rooted in human history, predating modern pharmaceuticals by millennia. Across diverse medical traditions, mostly plants but also microbes and animal-derived products have been formulated into powders, salves, extracts, and bandages for their therapeutic values in promoting tissue repair and infection control [4,11,12,13,14]. Extensive ethnopharmacological and preclinical knowledge discusses the effectiveness of natural products for wound healing. More recently, systematic reviews have reported that over 150 medicinal plants and their isolates have shown positive effects on wound closure, epithelialization, and infection control in animal or human studies [4,11], and antioxidant natural compounds help in the inflammatory stage of wound healing [15]. Additionally, a crucial aspect of natural products for wound healing is the increasing challenge posed by antibiotic-resistant wound pathogens since chronic wounds often contain multiple microbial species, with these organisms embedded in biofilms that protect them from both the body’s immune responses and pharmaceutical treatments. Notably, pathogens like *Staphylococcus aureus*, *Pseudomonas aeruginosa*, and *Enterococcus* spp. are particularly prevalent and difficult to eradicate [4,13]. Many natural product-derived agents exert multimodal antimicrobial effects by disrupting microbial membranes, interfering with quorum sensing, or inhibiting biofilm formation, thus offering alternative or adjunctive strategies to standard antibiotics [4,11,13]. Phytochemicals and plant extracts have demonstrated efficacy in modulating nearly every phase of the wound healing process, acting as antimicrobial agents, antioxidants, free radical scavengers, promoters of collagen production, stimulators of angiogenesis, and immune response modulators. Such bioactive compounds, including alkaloids, flavonoids, quinones, saponins, tannins, terpenoids, phenolic compounds, and essential oils, both target infectious agents and regulate the tissue regeneration required for healing [1,4,11,12,13,14,16]. When classified according to their mechanism of action in wound healing, according to a bibliographic study about wound healing by natural compounds published by Trinh and coworkers (2022), studies focus on anti-inflammation (with 67 + 7) (publications about compounds from plants and from animals, respectively, as will be mentioned hereinafter), antioxidant (58 + 5), antibacterial (24 + 4), collagen promotion (17 + 2), antimicrobial (12 + 5), pro-angiogenesis (13 + 3), antifungal (10 + 1), antiviral (7 + 2), antidiabetic (6 + 0), analgesic (5 + 0), and hemostatic (3 + 0) activities [16], with no mention of other sources of natural compounds for wound healing (such as microbials). Other reviews about natural compounds by natural products specifically focus on phytochemicals [4,11,13,14] or do not mention microbial sources [1,17].

Nevertheless, of special relevance to recent biomedical innovation is the microbiota residing within and upon plants, especially the endophytic microorganisms that inhabit internal plant tissues. Endophytes, which consist mainly of bacteria and fungi, have traditionally been considered neutral commensals, but research over the past two decades has dramatically re-shaped this view. Far from being passive, many endophytes engage in a mutualistic to symbiotic relationship with their host plant, providing adaptive advantages that are increasingly being recognized as fundamental to plant health, growth, and resilience [18,19,20,21,22,23,24]. Endophytes inhabit virtually every plant species examined to date, colonizing roots, stems, leaves, flowers, and seeds without causing overt pathogenicity [25,26,27]. Plants offer these microorganisms a protected ecological niche, metabolic resources derived from photosynthesis and primary metabolism, and a comparatively stable microclimate. In return, endophytes safeguard their host against pathogens via the secretion of antimicrobial and antifungal metabolites, promote growth via the synthesis of phytohormones, such as indole-3-acetic acid (IAA) and gibberellins, and help the plant adapt to biotic and abiotic stresses such as drought, salinity, cold, or exposure to toxic compounds by bolstering stress response pathways [19,22,23,24,28,29,30]. What distinguishes the interaction between plants and endophytes as symbiotic, rather than commensal, is the remarkable reciprocity of regulation and cross-communication. Plants possess the means to alter their internal chemical milieu to encourage or suppress specific endophytic strains, while endophytes dynamically regulate their own gene expression and secondary metabolism in response to plant-derived cues or environmental stressors. These molecular dialogues confer a high degree of ecological plasticity and mutual adaptive benefit [19,24,28].

Crucially, the ecological arms race between endophytes, their host plants, and external microbial or insect aggressors has driven endophytes to become prolific and versatile producers of bioactive secondary metabolites. The structural diversity and bioactivity spectrum of endophyte-derived compounds rival, and often surpass, that of their plant hosts [31,32,33]. These include alkaloids, flavonoids, phenolic acids, terpenoids, polyketides, exopolysaccharides, and a range of low-molecular-weight peptides and proteins, many of which are not present in the plant itself or are produced at substantially higher concentrations in endophytes. Ecological studies, as well as pharmacological screens, consistently demonstrate that endophytic strains isolated from medicinal or stress-prone plants are rich sources of compounds with antimicrobial, antioxidant, anti-inflammatory, and tissue regenerative capacities [34]. It has also become clear that certain high-value pharmaceutical agents, originally attributed to plants, in fact arise from or are substantially modulated by plant-associated endophytes [19,20,22,23,24,30]; the classic example being the anticancer agent taxol (paclitaxel), which was first commercialized from yew trees but was subsequently shown to be of endophytic origin [21].

The therapeutic arsenal offered by endophyte-derived secondary metabolites may be suitable to improve wound healing. The recent experimental literature demonstrates that endophyte extracts and purified components potentially possess the ability to modulate several processes related to phases of wound healing [18,20,35]. There are several reviews available in the literature where the pharmaceutical potential of compounds extracted from endophytic microorganisms is described; however, none of them specifically focuses on wound healing [18,36,37,38,39,40,41,42]. There is one review where the authors suggest using compounds from endophytic microorganisms for preparing bioactive wound dressings, but studies linking antimicrobial activities of compounds from endophytic microorganisms (Table 4 in Firoozbahr et al., 2023) with microorganisms associated with wound infections (such as *S. aureus*, *E. coli*, *P. aeruginosa*, etc.) were not specifically investigated in the context of wound healing [43]. Therefore, our objective in this work is to provide a review of compounds derived from endophytic microorganisms specifically aimed at wound healing. We conducted a literature search on scientific databases to identify primary research on compounds from endophytic microorganisms that are directly involved in wound healing pathways and were investigated in such a context. It is noteworthy that there is a limited number of studies specifically addressing the isolation and characterization of agents from endophytic microbial sources aimed at improving wound healing. This scarcity may be due to the early stage of this research field, the vast diversity of uninvestigated endophytic microorganisms, and the considerable untapped potential for discovering new therapeutic compounds. Consequently, this review provides a concise, albeit partial, summary of the potential of compounds from endophytic microorganisms in wound healing. Given our prior comprehensive review about the antimicrobial activities of compounds from endophytic microorganisms [37], the current investigation will pivot to delineate alternative mechanisms by which secondary metabolites from endophytic microorganisms contribute to the multifaceted process of wound healing. Thus, the discussion of antimicrobial properties will be selectively incorporated, focused mainly on studies where such characteristics are explicitly investigated within the context of wound repair and tissue regeneration. The ultimate aim of this review relates to a practical aspect of ecopharmacognosy. It highlights the ongoing effort to shift the production of commercially important secondary metabolites to methods that are more controlled, environmentally sustainable, consistently reproducible, and economically feasible, with endophytic microorganisms showing promise as potential production systems.

## 2. Pharmacological Potential of Compounds from Endophytic Microorganisms in Wound Healing

**Figure 1:**
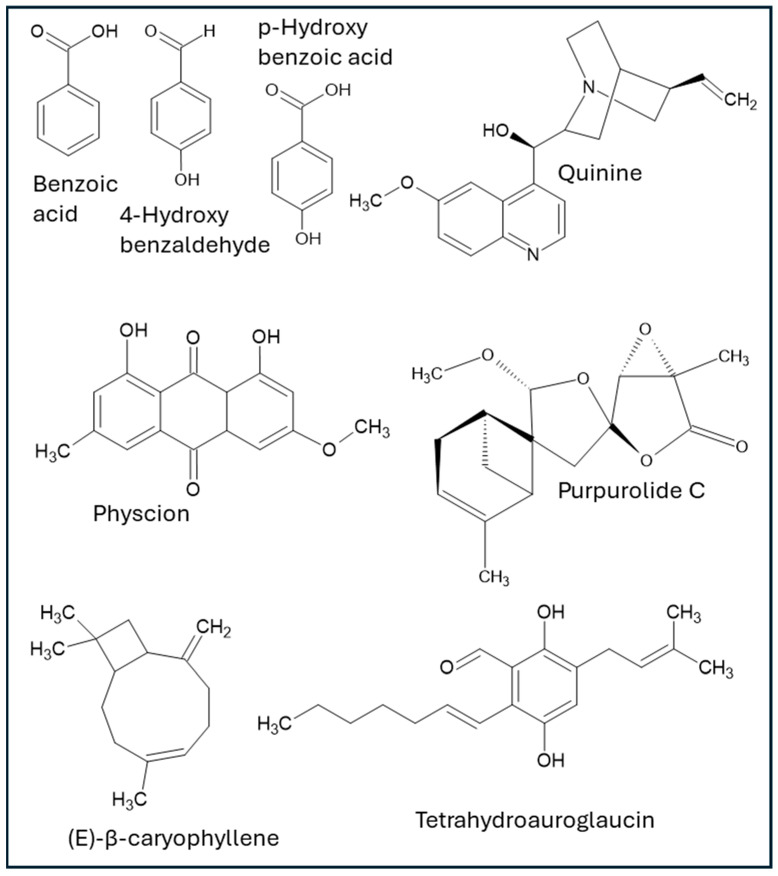
Structures of specific compounds derived from endophytic microorganisms with effects on wound healing.

Endophytic microorganisms associated with medicinal and non-medicinal plants are significant sources of diverse bioactive compounds, which have demonstrated activities directly relevant to wound healing. These include, but are not limited to, aromatic acids, phenolics, anthraquinones, terpenoids, glycosides, polysaccharides, alkaloids, peptides, polyketides, saponins, and exopolysaccharides. The following chemical families of compounds are documented within the analyzed literature (structures of bioactive compounds from endophytes with wound healing properties are depicted in Figure 1, and summary of activities is shown in Table 1).

### 2.1. Phenolic Compounds

Classification was conducted according to [44]. Phenolic compounds have demonstrated wound healing activity in both in vivo and in vitro models through various mechanisms. These include boosting antioxidant defenses, lowering oxidative stress, adjusting inflammatory responses, speeding up healing times, decreasing infection rates, and promoting tissue regeneration [1,45,46]. Specific works about the wound healing effect of phenolic compounds from endophytic microorganisms may be found in the literature [22,47,48]. There are also several studies where compounds from endophytic microorganisms possess a biological activity that may indicate potential for wound healing, and although the direct application for such purpose has not been directly established, there are some other studies where the same compound (s), isolated from different sources, have been indeed linked to wound healing. We are also enlisting two examples of such cases [47,49].

Benzoic acid is produced by the *Neurospora crassa* strain SSN01 and is isolated from *Lycium shawii* leaves from Egypt. Benzoic acid demonstrated potent antimicrobial activity against wound-relevant Gram-positive and Gram-negative bacteria (including drug-resistant *Staphylococcus aureus* and *Pseudomonas aeruginosa*) with the minimum inhibitory concentration (MIC) ranging between 256 μg/mL and 1024 μg/mL against reference strains and clinical isolates of MRSA and MSSA from infected wounds, as well as significant inhibition of biofilm formation. Additionally, in vivo histopathological examination revealed that benzoic acid accelerated wound contraction and epithelialization in rat excision models [48].

Taher et al. (2023) investigated the antimicrobial activities of secondary metabolites from the endophytic fungus *Phyllosticta fallopiae* L67, isolated from *Aloe vera*, in the context of diabetic wounds. They identified an active fraction containing biologically important metabolites. The results showed that the dichloromethane extract exhibited the most significant antimicrobial activity, with inhibition zones ranging from 11.33 to 38.33 mm. The minimal inhibitory concentrations (MICs) and minimal lethal concentrations (MLCs) of the dichloromethane extract ranged from 78.13 to 2500.00 µg/mL and 625.00 to 5000.00 µg/mL, respectively. Additionally, the analysis of the bioactive fraction using the UPLC-QTOF-MS/MS method revealed the presence of flavones, stilbenes, flavanonols, isoflavonoids, phenolic glycosides, and phenol derivatives. The extract also inhibited more than 82% of biofilm formation [22].

4-hydroxybenzaldehyde is a compound that was isolated from the endophytic fungus *Epicoccum nigrum* (strain RAM10-1) from fresh leaves of *Entada abyssinica*, a plant collected in Cameroon by Dzoyem et al. (2017) [47]. The research demonstrated that 4-HBA possesses notable biological activities, particularly antibacterial and antioxidant properties. In terms of its antibacterial effects, 4-HBA showed varying levels of minimum inhibitory concentrations (MICs) against several bacterial strains. Specifically, it was effective against *Bacillus cereus* and *Escherichia coli* at an MIC of 25 µg/mL. Against *Staphylococcus aureus* and *Pseudomonas aeruginosa*, the MIC was 50 µg/mL, and against *Enterococcus faecalis*, it was 100 µg/mL. Regarding its antioxidant capabilities, 4-HBA exhibited considerable free radical scavenging activity, since its IC_50_ value was 38.43 µg/mL in the DPPH assay and 49.45 µg/mL in the ABTS assay. Furthermore, its antioxidant power was measured at 12.12 µmol FeSO_4_/g using the FRAP assay. The study also assessed the cytotoxicity of 4-HBA against different cell lines. The IC50 values, indicating the concentration at which 50% of cells are inhibited, were 38.33 µg/mL for Vero cells, 31.87 µg/mL for THP-1 cells, and 48.62 µg/mL for RAW 264.7 cells. Other compounds, including beauvericin, indole-3-carboxylic acid, and quinizarin, were also investigated and showed promising results regarding antioxidant, antibacterial, and cytotoxicity [47]. However, although 4-HBA was not specifically tested for wound healing in the aforementioned study, this compound has been shown to have an active role in wound healing by promoting keratinocyte cell migration and invasion by increasing focal adhesion kinase and Src activity and by promoting wound healing and re-epithelization in an in vivo animal model, among other mechanisms [50].

p-hydroxybenzoic acid and ferulic acid were isolated from *Aspergillus* sp., endophytes of *Moringa oleifera* from Nigeria. At a concentration of 250 µg/mL, ferulic acid demonstrated remarkable antioxidant activity, achieving an inhibition rate of 90.4%. In comparison, p-hydroxyphenyl acetic acid resulted in an inhibition of 35.4%. Additionally, ferulic acid displayed some antifungal activity at 500 µg/mL against *A. niger* [49]. Although the aforementioned study was not specifically about wound healing, it has been reported that these compounds have promoted wound healing in in vivo models [46].

### 2.2. Anthraquinones

Anthraquinones are a diverse group of naturally occurring phenolic compounds that are primarily derived from the 9,10-anthraquinone structure. This aromatic organic compound, known as anthracenedione or dioxoanthracene, has the chemical formula C_14_H_8_O_2_. Although several isomers exist, the term generally refers to 9,10-anthraquinone (IUPAC name: 9,10-dioxoanthracene), characterized by the positioning of the keto groups on the central ring [51]. Anthraquinones are widely distributed in nature and are produced primarily by plants and microorganisms. These compounds display a broad range of biological activities. While there are reviews covering the bioactive properties of endophytic fungal anthraquinones and their analogs, as well as their antibacterial, antifungal, anti-inflammatory, and antioxidant effects, which are mechanisms relevant to wound healing, there is no direct reference to their specific application in wound treatment [52,53]. However, to display the wound healing potential of anthraquinones or derivatives derived from endophytic microorganisms, we can reference research that connects compounds produced by these endophytes to wound healing properties documented in other studies. Such an example establishes an indirect link between endophytic metabolites and their possible therapeutic applications in wound treatment.

Elawdy and coworkers (2023) isolated physcion, a dihydroxyanthraquinone, from *Aspergillus versicolor*, an endophytic fungus from *Juncus rigidus.* The authors revealed that physcion demonstrated significant anti-inflammatory effects by inhibiting both COX-2 and LOX-1 enzymes, with respective IC_50_ values of 43.10 µg/mL and 17.54 µg/mL. Furthermore, it displayed substantial anticholinesterase activity (86.9 ± 1.21%). In addition, it showed robust antioxidant capabilities, as confirmed by its effectiveness in neutralizing various reactive species, including DPPH, ABTS, and O_2_ radicals, as well as nitric oxide (NO), and by its capacity to prevent lipid peroxidation [54]. Physcion, in another study using an in vivo model, has been shown to effectively accelerate diabetic wound healing by promoting collagen regeneration and epidermal repair [55].

### 2.3. Alkaloids

Alkaloids, a structurally diverse group of nitrogen-containing natural compounds, are renowned for their wide spectrum of pharmacological activities and important therapeutic potential. These activities include, but are not limited to, anticancer (e.g., vinblastine, vincristine, camptothecin), antimicrobial, anti-inflammatory, analgesic, and neuropharmacological effects, making them a cornerstone in drug discovery and traditional medicine. Endophytic fungi have emerged as a prolific source of novel and known alkaloids, some of which were previously thought to be exclusive to their host plants. Although the literature extensively documents these varied biological actions and the potential of endophytic alkaloids in treating a range of diseases, specific, in-depth studies focusing directly on the wound healing capabilities of alkaloids derived from these microorganisms are not, to the author’s best knowledge, reported in the scientific literature [56,57]. However, given their well-established antibacterial, anti-inflammatory, and, in some cases, cell-proliferation-modulating properties, there is a strong underlying rationale to explore their potential in promoting and accelerating wound repair processes. This suggests a promising, yet underexplored, avenue for future research to technically evaluate and harness alkaloid bioactivity for wound management applications. It is worth noting that there are studies where alkaloids have been linked to the inhibition of wound healing, as is the case with camptothecin (CPT), a monoterpene alkaloid that has been isolated from *Entrophospora infrequens*, an endophyte of *Nothapodytes foetida* [58], and from *Aspergillus terreus* [59], an endophyte of *Catharanthus roseus*, among other sources [56]. After 24 h, the CPT-treated UO-31 cells showed approximately 55.5% wound closure, while the untreated cells achieved about 97% closure [59]. Another case is vinblastine, an indole alkaloid produced by the endophytic fungi *Curvularia verruculosa* from the leaves of *Catharanthus roseus* [60], which has been reported as an inhibitor of wound healing [61]. Nevertheless, there are few studies that may, albeit indirectly, link the potential of alkaloids for wound healing.

Quinine, a widely known anti-malarial drug normally extracted from the bark of the cinchona tree, has also been shown to be isolated from *Colletrotrichum* spp., an endophyte from *Cinchona calisaya* Wedd. [62]. This compound has been known to be an effective remedy for treating infected wounds since 1916, as referred to by Lt.Col. P.H. Falkner, R.A.M.C. [63], with more modern studies where its use is being reported for skin-related diseases [64,65].

### 2.4. Terpenoids

Terpenoids, a vast and structurally varied class of natural products, exhibit a wide array of bioactivities, including anticancer, anti-inflammatory, antibacterial, antiviral, and anti-malarial effects, positioning them as molecules with considerable therapeutic potential. They also show promise as insect resistance agents, immunoregulators, antioxidants, anti-aging compounds, and neuroprotective agents. Despite the extensive research cataloging these diverse biological effects, specific investigations into the direct application of terpenoids from endophytic fungi for wound healing are seldom detailed in the literature [66]. Nevertheless, their established antibacterial, anti-inflammatory, and antioxidant properties inherently suggest a strong potential for facilitating wound repair and tissue regeneration, warranting further targeted research in this area. It is worth mentioning, though, that taxol, one of the first drugs from endophytic microorganisms, a diterpenoid widely distributed among several species of endophytes [56], is known for increasing the chemotherapy-related complications of wound healing [67]. There is, to the author’s knowledge, one study where terpenoids from endophytes have been positively associated with wound healing [68]. Nevertheless, the effect of terpenoids in wound healing is well-documented [69]; thus, in order to illustrate the potential of terpenoids from endophytic microorganisms for wound healing, there may be also cited as an example a study that links compounds reported as being produced by endophytic microorganisms that have been reported elsewhere as linked to wound healing [70].

In a study by Liu and coworkers (2023), purpurolide C (PC), a sesquiterpene lactone isolated from *Penicillium porpurogenum*, was found to promote wound healing through the inhibition of inflammatory macrophage activation by inhibiting signaling processes involved in the lipopolysaccharide (LPS)-induced macrophage activation pathway. PC was found to be a competitive antagonist of LPSs by inhibiting TLR4-MD2 dimerization and MYD88 phosphorylation, which led to the inhibition of the NF-κB pathway, thus dampening the pro-inflammatory cytokines, shifting the pro-inflammatory macrophages to an anti-inflammatory population, and addressing one of the mechanisms of diabetic wound chronicity. In vitro and in vivo studies were carried out, and PC was regarded as a novel biomaterial with potential for treating diabetic wound healing [68].

(E)-β-caryophyllene, a sesquiterpene normally found in several herbs and spices, was isolated from a culture of an endophytic fungus, a species of *Cladosporium*, which colonizes black poplar (*Populus nigra*) leaves [70]. This compound has a well-documented wound healing effect, mainly through re-epithelialization and anti-inflammatory mechanisms (as a ligand of cannabinoid receptor 2 [CB2], which is involved in the inflammatory response). Additionally, other mechanisms have been described, including evidence from in vivo models [71]. It is worth mentioning that Walther and coworkers actually found sesquiterpene synthases in *Cladosporium* and were able to successfully identify other terpenes, such as E)-β-farnesene, β-himachalene, and β-bisabolene, among various other partially identified terpenes.

### 2.5. Proteins and Enzymes

There are several papers and reviews where the potential of endophytic microorganisms for producing proteolytic enzymes is described [72,73,74]. Although, to the author’s knowledge, no investigation was carried out regarding wound healing, and there was no specific protease reported in endophytic microorganisms that could be linked to specific studies regarding wound healing; however, the effect of proteases in pathological situations is still documented. They may be involved in cellular growth and development, the production and breakdown of collagen, the formation and resolution of perivascular fibrin, and the clearing of necrotic tissue post-inflammation [75]. However, since a relatively small group of enzymes handles these varied tasks, forecasting the impact of introducing synthetic proteolytic enzymes from endophytic microorganisms to a wound site is complex and should be investigated in the future.

### 2.6. Polysaccharides/Glycoconjugates

Recent research highlights that both endophytic bacteria and fungi are proficient at producing exopolysaccharides (EPSs) [41]. An existing review article summarizes the latest developments concerning EPSs sourced from endophytes, encompassing their methods of production, techniques for isolation and purification, detailed structural characterization, natural physiological functions, and observed biological activities. These EPS molecules can range structurally from simple, linear homopolysaccharides to highly branched heteropolysaccharides. Their novel structures are associated with several beneficial biological effects, such as antioxidant, antitumor, anti-inflammatory, anti-allergic, and prebiotic properties. Although the aforementioned review does not directly address the topic of wound healing, some of the described biological activities may contribute to it [76]. Nevertheless, two distinct studies have specifically investigated the potential of EPSs from endophytic microorganisms in promoting wound healing.

The extracellular polysaccharides produced by the endophytic fungus *Talaromyces purpureogenus* demonstrated significant in vitro wound healing activity and cellular antioxidant properties in the human embryonic kidney (HEK293) cell line [77]. This wound healing activity may be attributed to the oligosaccharides present within the polysaccharides. Specifically, oligosaccharides, particularly the 1,3-D-glucan linkers, are known to effectively stimulate cells to produce cytokines, thereby accelerating cell growth and proliferation [78].

The exopolysaccharide extract from *Papiliotrema terrestris* PT22AV, isolated from *Olea europaea*, demonstrated notable wound healing activity (percentage of wound closure of 99.2%) at a concentration of 10 mg/mL after 14 days during in vivo studies carried out in Wistar rats, where comparison was performed with both negative (no treatment) and positive (phenytoin 1% cream) controls. Additionally, the exopolysaccharide extract exhibited antibacterial activity by over 80% inhibition against *Escherichia coli*, *Staphylococcus aureus*, and *Staphylococcus epidermidis* at a concentration of 2 mg/mL while also showing cytocompatibility with human fibroblast and macrophage cell lines. The composition of the exopolysaccharide was determined to consist of 97% mannose and 3% glucose in molar percentages. Therefore, *P. terrestris* PT22AV represents a promising source of compounds with potential for skin wound healing [35].

### 2.7. Other Compounds/Mixtures

The ethanolic extract of the endophytic fungus *Penicillium amestolkiae* elv609, obtained from *Orthosiphon stamineus* Benth, was tested against various clinical pathogens, including four Gram-positive bacteria (*Bacillus cereus*, *Bacillus coagulans*, *Streptococcus* sp., and *Staphylococcus aureus*), four Gram-negative bacteria (*Escherichia coli*, *Proteus mirabilis*, *Yersinia* sp., and *Pseudomonas aeruginosa*), and two yeasts (*Candida albicans* and *Candida utilis*) isolated from diabetic wounds. The extract exhibited significant antimicrobial activity against four bacteria (*Streptococcus* sp., *Bacillus cereus*, *Escherichia coli*, and *Pseudomonas aeruginosa*) and one yeast (*Candida utilis*), with minimal inhibitory concentrations (MICs) ranging from 6.25 to 12.5 mg/mL. Gas chromatography–mass spectrometry (GC-MS) analysis of the extract identified 6-octadecenoic acid as the main constituent, followed by Dihydro-2-methyl-3(2H)-furanone and n-Hexadecanoic acid [79]. Diethyl phthalate was also identified, but this compound has been associated with bleeding from GC septa; thus, its presence in the chromatogram could be a contamination [80]. Furthermore, when the fungal culture was treated with textile materials, the growth of bacteria was inhibited by up to 99.9% following the application of the textile. After 50 washes with commercial detergent, the textile continued to demonstrate effective antimicrobial activity [79].

Secondary metabolites of the endophytic fungus *Paecilomyces* sp. AUMC 15,510 isolated from healthy stem samples of the Egyptian medicinal plant *Cornulaca monacantha* showed promising wound healing activity and enhanced wound closure on earthworms (*Lumbricus castaneus*), which is a feasible and plausible model that mimics human skin. In addition, the ethyl acetate crude extract of *Paecilomyces* sp. exhibited significant antimicrobial activity against six human microbial pathogens. Therefore, the endophytic fungus *Paecilomyces* sp. (AUMC 15510) could be a sustainable source of a potential therapeutic target for wound management and antimicrobial agents [20]. Within those compounds extracted with ethyl acetate, there were phenolics and flavonoids, including naringenin, caffeic acid, and quercetin, previously reported as possessing antioxidant, anti-inflammatory, antimicrobial, and wound healing activities [81,82,83]. In the extract, other compounds where wound healing activity had also been previously reported were identified as follows:

- The polyphenol 4-chloro-3,5-dimethylphenol; alkanes and alkenes; cetene and pentacosane; the long-chain fatty alcohol 1-eicosanol; and the long-chain fatty acid erucic acid, all of which have exhibited antimicrobial, skin disinfection, and wound disinfection effects [84,85,86,87,88].
- The fatty acid esters 9,12-Octadecadienoic acid (Z,Z)-methyl ester and 9-Octadecenoic acid, methyl ester (E), and the isoflavone daidzein, all of which exhibit anti-inflammatory, antibacterial, skin repair, and wound healing effects [89,90,91,92].

The ethyl acetate extract of the endophytic fungus *Preussia africana*, which belongs to the family *Sporormiaceae* and is isolated from *Aloe vera*, demonstrated a strong in vitro wound healing effect (by evaluating cell motility of the MCF-7 cells) at a concentration of 500 μg/mL (42.6% at 48 h) compared to the positive control. Additionally, the crude extract of this endophytic fungus exhibited cytotoxic activities against several cancer cell lines, including HeLa (cervical cancer), Hep G2 (liver cancer), MCF-7 (breast cancer), A549 (lung cancer), LN-229 (glioblastoma), A-431 (skin cancer), and the kidney cell line HEK 293T, at a concentration of 50 μg/mL. It is also worth noting that individual compounds were not identified in the study, although the ethyl acetate extract was screened for alkaloids, flavonoids, phenols, tannins, terpenoids, steroids, and saponins [93].

Vasarri et al. (2022) isolated six compounds from the endophytic fungus *Eurotium chevalieri* MUT 2316, obtained from *Grantia compressa*, and examined the wound healing activities of prenylated benzaldehyde derivatives that had been reported as purified in larger quantities: tetrahydroauroglaucin and dihydroauroglaucin. Wound closure was significantly more rapid in cells treated with tetrahydroauroglaucin, achieving complete closure within 24 h compared to cells treated with dihydroauroglaucin, despite the structural similarities between the two compounds. Additionally, both molecules displayed no cytotoxicity according to the MTT assay [23].

An ethyl acetate (EtOAc) extract of *Penicillium rubens* (EPR), an endophyte isolated from the leaves of *Cucumis sativus* L., was determined to possess multifaceted biological activities. Antibacterial tests showed that EPR (1000 µg/mL in ethyl acetate) created a 24 mm inhibition zone against *Pseudomonas aeruginosa* (ATCC 27853) via the agar disc diffusion method. Its MIC against 20 clinical P. aeruginosa isolates ranged from 128 to 1024 µg/mL, determined by broth microdilution. The extract also demonstrated potential anti-inflammatory effects through an assay involving lipopolysaccharide (LPS)-stimulated WI38 cells. While LPS (20 µg/mL) stimulation increased pro-inflammatory cytokine TNF-α gene expression in WI38 cells by approximately 3.08-fold (±0.03), subsequent EPR treatments significantly lowered this to about a 1.09-fold increase (±0.02, *p* < 0.0001), as per qRT-PCR analysis. Moreover, using in vitro wound healing assays, EPR significantly enhanced wound closure percentages. After 24 h, EPR-treated wounds closed by 66.64% (±5.61) compared to 13.79% (±3.98) in controls. By 48 h, these figures were 99.94% (±0.05) for EPR and 83.37% (±0.05) for controls [94].

Secondary metabolites from two endophytic actinomycetes, *Streptomyces parvulus* GloL3 and *Streptomyces lienomycini* SK5, isolated from Indian medicinal herbs *Globba marantina* L. and *Selaginella kraussiana*, showed wide-ranging biological activity. The ethyl acetate (EA) extract from SK5 demonstrated antimicrobial properties against a variety of human pathogens, including drug-resistant *Staphylococcus aureus* (MRSA), *Candida tropicalis*, and *C. albicans*. It was effective at a minimum microbicidal concentration (MMC) ranging from 50 to 300 µg/mL. Furthermore, this extract successfully treated MRSA-infected wounds in Swiss albino mice. The mechanism of action involves disrupting the cell membranes of these pathogens, leading to the leakage of essential biomacromolecules like nucleic acids, proteins, and potassium ions. Additionally, the extract inhibited crucial housekeeping enzymes vital for the pathogens’ cellular respiration. On the other hand, *Streptomyces parvulus* GloL3 exhibited antioxidant capabilities against various free radicals (DPPH, ABTS, FRAP, and H_2_O_2_), with IC50 values of 21.18 ± 0.33, 43.58 ± 0.91, 88.24 ± 1.24, and 111.03 ± 6.42 µg/mL, respectively. It also enhanced the enzymatic antioxidant levels in treated peritoneal macrophage cells from Swiss albino mice. Analysis of the EA extracts from both GloL3 and SK5 revealed the presence of compounds such as bactobolin, actinobolin, 5-(2-aminoethyl)-1 H imidazole-2-carbaldehyde, isovaleric acid, fulvic acid, phenol, 4-[2-(methylamino) ethyl]-, eicosanoic acid, and heptadecanoic acid [95].

In a study carried out by the authors of a review on the potential of compounds from endophytic microorganisms as antibacterial additives in bioactive wound dressings, thirty-two endophytic fungi, isolated from thirteen unique Australian native plant species, were studied for their antibacterial activity against *S. aureus*. Fungal culture filtrate extracts using ethyl acetate (EtOAc) showed inhibitory action against both methicillin-sensitive *S. aureus* (ATCC 25923) and MRSA (M180920), with a minimum inhibitory concentration (MIC) of 78.1 µg/mL for each. Additionally, cytotoxicity studies using the brine shrimp lethality test demonstrated low cytotoxicity of up to 6 × MIC (25% mortality rate), with an LC50 value of 639.1 µg/mL. However, no specific compounds were reported in the study [96].

A mixture of compounds extracted from *Umbelopsis* sp. TM01, an endophytic fungus isolated from the fruiting body of *Tricholoma matsutake* (a mycorrhizal fungus), was evaluated as an alternative to the skin care effects of *T. matsutake* itself. Metabolomic studies were performed by UPLC-Q-TOF-MS/MS, and several compounds were identified, including carbohydrates and carbohydrate conjugates, organic acids and derivatives, lipids and lipid-like molecules, alkaloids and derivatives, benzenoids, phenylpropanoids, and polyketides. In a cytotoxicity assay, it was determined that *Umbelopsis* sp. TM01 extract (UFE) had no toxicity to human dermal fibroblasts at the tested concentrations. In a cell scratch assay, which is an in vitro model designed by the authors to study the effects of UFE on cell migration and repair, the results indicated a concentration-dependent effect on repair rates. The authors concluded that UFE exhibited more potent anti-tyrosinase activity, greater anti-wrinkle efficacy, and a comparable wound healing effect to that of *Tricholoma matsutake* extract (TME) [97].

**Table 1:** Compounds isolated from endophytic microorganisms with effects on wound healing, investigated specifically in that context.

Endophyte	Host	Secondary Metabolites	The Effects on Wounds	References
*Paecilomyces* sp. AUMC 15510	*Cornulaca monacantha*	Ethyl acetate crude extract	Wound closure activity	[20]
*Neurospora crassa* SSN01	*Lycium shawii*	Benzoic acid	Burn wound infections	[48]
*Talaromyces purpureogenus*		Extracellular polysaccharides	Wound healing activity and cellular antioxidant	[77]
*Preussia africana*	*Aloe vera*	Ethyl acetate crude extract	In vitro wound healing and anticancer effect	[93]
*Phyllosticta fallopiae* L67	*Aloe vera*	Dichloromethane extract	Diabetic wound healing	[22]
*Penicillium amestolkiae* elv609	*Orthosiphon stamineus Benth*	Ethyl acetate crude extract	Diabetic wound healing	[79]
*Papiliotrema terrestris* PT22AV	*Olea europaea*	Exopolysaccharide	Wound closure activity	[35]
*Eurotium chevalieri* MUT 2316	*Grantia compressa*	Tetrahydroauroglaucin and dihydroglaucin	Wound closure effect	[23]
*Penicillium porpurogenum*	*-*	Purpurolide C	Diabetic wound healing	[68]
*Penicillium rubens*	*Cucumis sativus* L.	Ethyl acetate crude extract	Wound closure effect	[94]
*Streptomyces parvulus GloL3 and Streptomyces lienomycini SK5*	*Globba marantina* L. and *Selaginella kraussiana*	Ethyl acetate crude extract	Antimicrobial and antioxidant	[95]

## 3. Critical Analysis and Translational Challenges

Using the GRADE framework [98], all 18 studies retrieved start as ‘low’ quality because they are preclinical. Additionally, the risk of bias, imprecision, and indirectness further downgrades 15 of them to ‘very low’. Only four studies include both in vitro mechanistic and in vivo efficacy data, warranting a ‘low’ rating (Table 2).

Regarding the quantitative assessment of the evidence, of the 18 studies reviewed, only 14 directly investigated the wound healing properties of endophyte-derived compounds (or extracts). The majority of the investigated studies (>60%) dealt with extracts or mixtures of compounds; thus, although in some cases the chemical composition was reported, it is still not possible to link the evidence on wound healing to any specific compounds from those studies. Only four studies progressed to in vivo models, and no studies advanced to clinical trials.

Regarding methodological limitations, there is a lack of standardization since studies employ diverse wound models (earthworms, cell scratch assays, rodent excision models), making cross-study comparisons difficult. Most studies report phenomenological observations without elucidating molecular mechanisms; thus, there is an incomplete mechanistic understanding. In none of the review studies are bioavailability concerns considered since no pharmacokinetic properties, stability, or delivery methods are being addressed. No large animal or human data are available, limiting extrapolation due to inter-species differences in skin architecture, immune response, and microbiome composition [99,100]. Additionally, the influence of host plant species, geographical location, and cultivation conditions on metabolite production remains unexplored.

To bridge these gaps and ultimately bring endophyte-derived compounds into clinical practice for wound healing, we believe that several research priorities should be addressed, such as (1) the development of standardized assays aimed at assessing potential wound healing properties of specific endophyte metabolites; (2) comparative efficacy studies against current clinical treatments to contextualize their therapeutic potential; (3) further investigations into specific mechanisms and structure–activity relationships, which will be essential for optimizing lead compounds; and (4) efforts should also focus on developing sustainable production methods and addressing the formulation and delivery challenges unique to wound healing applications.

## 4. Conclusions

Endophytic microorganisms represent a promising and largely unexplored reservoir of novel bioactive compounds with direct relevance to wound healing. The diverse chemical families produced by these endophytes, including phenolic compounds, terpenoids, alkaloids, and polysaccharides, among others, have demonstrated mechanisms pertinent to various stages of wound repair, such as antioxidant, anti-inflammatory, and antimicrobial actions, as well as promoted tissue regeneration. Although direct research focusing on endophyte-derived compounds for wound healing is still in its nascent stages, the existing evidence strongly suggests their significant therapeutic potential. The practical implication is clear: endophytic secondary metabolites can provide sustainable, cost-efficient alternatives to current wound therapies, particularly at a time of rising antibiotic resistance and demand for innovation in chronic wound care. To fully realize this potential, immediate priorities include (1) standardizing models for wound healing assessment, (2) isolating and chemically characterizing individual active compounds from endophyte extracts, (3) elucidating precise molecular and cellular repair pathways engaged by these metabolites, and (4) initiating comparative and translational studies, including formulation optimization and in vivo pharmacokinetics, in larger animal models. Ultimately, integrating endophyte-derived compounds into advanced biomaterials and clinical therapeutics could help boost the landscape of wound management and tissue regeneration, particularly in diabetic and other chronic wounds. Future research must bridge laboratory discoveries and scalable, safe applications to unlock the promise of endophytes for next-generation wound care solutions.

## Figures and Tables

**Table 2:** Compounds isolated from endophytic microorganisms with effects on wound healing, investigated specifically in that context. Abbreviations: DICM: dichloromethane. EtOH: ethanol. EtAc: ethyl acetate. EPS: extracellular polysaccharide. PDB: potato dextrose broth. GRADE: Recommendation Assessment, Development, and Evaluation. D: direct evidence (compound investigated in the context of wound healing). I: indirect evidence (compound reported in endophytes but not investigated in the context of wound healing, and an explanation can be found in Section 2 of the present manuscript).

Type of Compound	Compound/Extract	Reference	D/I	In Vivo Studies Regarding Wound Healing?	Reference for In Vivo Model	GRADE Rating
Phenolic	Benzoic acid	[48]	D	Yes		Low
	DICM extract	[22]	D	No		Very Low
	4-Hydroxybenzaldehyde	[47]	I	Yes (but indirect)	[50]	Very Low
	p-hydroxybenzoic acid	[49]	I	Yes (but indirect)	[46]	Very Low
Anthraquinones	Physcion	[54]	D	Yes (but indirect)	[55]	Very Low
Alkaloids	Quinine	[62]	I	Yes (but indirect)	[65]	Very Low
Terpenoids	Purpurolide C	[68]	D	Yes		Low
	(E)-β-caryophyllene and others	[70]	I	Yes (but indirect)	[71]	Very Low
Polysaccharides	EPS	[77]	D	No		Very Low
	EPS	[35]	D	Yes		Low
Other	EtOH extract	[79]	D	No		Very Low
	EtAc extract	[20]	D	Yes		Low
	EtAc extract	[93]	D	No		Very Low
	Tetrahydroauroglaucin	[23]	D	No		Very Low
	EtAc extract	[94]	D	No		Very Low
	EtAc extract	[95]	D	No		Very Low
	EtAc extract	[96]	D	No		Very Low
	PDB extract	[97]	D	No		Very Low

## Data Availability

No new data were created or analyzed in this study. Data sharing is not applicable to this article.

## Abbreviations

The following abbreviations are used in this manuscript:

ECM	Extracellular Matrix
EPS	Extracellular Polysaccharide
HBA	Hydroxybenzaldehyde
MIC	Minimum Inhibitory Concentration
UPLC	Ultra-High-Performance Liquid Chromatography
TOF	Time of Flight
EtOH	Ethanol
EtAc	Ethyl Acetate
PDB	Potato Dextrose Broth
DICM	Dichloromethane
D	Direct Evidence
I	Indirect Evidence
GRADE	Recommendation Assessment, Development, and Evaluation
MS	Mass Spectrometry
DPPH	2,2-Diphenyl-1-Picrylhydrazyl
TPH	Human monocytic cell line derived from an acute monocytic leukemia patient
ABTS	2,2’-Azino-Bis(3-Ethylbenzothiazoline-6-Sulfonic Acid
